# Genito-Urinary and Venereal Diseases

**Published:** 1893-02

**Authors:** 


GENITO-URINARY AND VENEREAL DISEASES.—A Manual for Students and Practitioners; by Charles H. Chetwood, M. D,, Visiting Surgeon Demilt Dispensary, Department of Surgery and Genito-Urinary Diseases, New York. Philadelphia, Lea Brothers & Co. Price $1.00.

THIS little work by Dr. Chetwood contains many points valuable alike to student and practitioner. Each subject is dealt with in a lucid manner, is well illustrated and will be found a very convenient reference book when an exhaustive work is not desired. C. E. J.

